# Meta-analysis of the cardioprotective effect of sevoflurane versus propofol during cardiac surgery

**DOI:** 10.1186/s12871-015-0107-8

**Published:** 2015-09-24

**Authors:** Feng Li, Yuan Yuan

**Affiliations:** 1 Department of Anesthesia, First People’s Hospital, Yancheng, Jiangsu Province 224000 China; 2 Department of Otolaryngology, First People’s Hospital, Yancheng, Jiangsu Province 224000 China

**Keywords:** Sevoflurane, Propofol, Cardiac surgery, Cardioprotective effect, Meta-analysis

## Abstract

**Background:**

To evaluate the cardioprotective effects of sevoflurane versus propofol anesthesia in patients undergoing cardiac surgery.

**Methods:**

Studies were retrieved through searching several databases. Study quality was evaluated by Jadad scale. Meta-analysis was performed with RevMan5.0 software. Publication bias was tested by funnel plot.

**Results:**

As a result, 15 studies were included. Compared with propofol, sevoflurane anesthesia significantly improved postoperative (WMD (weighted mean difference) = 0.62, 95% CI: 0.33 to 0.92; P < 0.0001) and postoperative 12 hour cardiac index (WMD = 0.18, 95% CI: 0.03 to 0.33; P = 0.02), postoperative cardiac output (WMD = 1.14, 95% CI: 0.74 to 1.54; P < 0.00001), and reduced postoperative 24 hour cardiac troponin I concentration (WMD = -0.86, 95% CI:-1.49 to -0.22; P = 0.008), postoperative inotropic drug usage (OR (odds ratio) = 0.31, 95% CI: 0.22 to 0.44; P < 0.00001), vasoconstrictor drug usage (OR = 0.30, 95% CI:0.21 to 0.43; P < 0.00001), ICU stay (WMD = -15.53, 95% CI: -24.29 to -6.58; P = 0.0007) and a trial fibrillation incidence (OR = 0.25, 95% CI: 0.07 to 0.85; P = 0.03). However, no significant differences were found in other indexes. Subgroup analysis indicated the similar results.

**Discussion:**

The sevoflurane-induced cTnI reduction is associated with lower incidence of late adverse cardiac events, accounting for its roles in cardiac protection. Several limitations existed such as the small sample size and the lack use of blind design.

**Conclusions:**

Sevoflurane may exhibit a more favorable cardioprotective effect during cardiac surgery than propofol.

## Background

Myocardial injury is a common complication in patients undergoing cardiac surgery, which can result in delayed recovery, organ failure, increased hospital length of stay, and mortality [1, 2]. To protect the myocardium from injury related to cardiac surgery, several approaches have been postulated, such as inhalation anesthetic preconditioning [3].

Volatile anesthetics have been suggested to contribute to myocardial protection through a preconditioning effect on the myocardium. The mechanisms involved in the protective effect of volatile anesthetic regimens are opening of mitochondrial KATP channels, activation of p38 mitogen-activated protein kinase, and an increase in mitochondrial reactive oxygen species. All these mechanisms account for decreased cytosolic and mitochondrial calcium loading [4–6]. A meta-analysis showed that volatile anesthetics, including sevoflurane, have beneficial effects on reducing morbidity and mortality, and thus play a cardioprotective effect on patients after cardiac surgery [7]. Intravenous anesthetics, such as propofol, are also reported to have a cardioprotective effect. This includes markedly decreasing the size of myocardial infarcts, lowering troponin release, and decreasing the rate of mortality after cardiac surgery [6, 8, 9]. A more recent study provided evidence that sevoflurane provides slightly better protection of the mitochondrial outer membrane than propofol in patients undergoing coronary artery bypass grafting (CABG) surgery with cardiopulmonary bypass [10]. However, which anesthetic is more favorable after cardiac surgery is controversial [11–13]. Recently published studies might provide additional information about the clinical outcomes of sevoflurane and propofol. Therefore, the two drugs’ cardioprotective effect on patients should be re-evaluated using powerful statistical analysis tools. Therefore, we performed a meta-analysis to compare the cardioprotective effects of sevoflurane and propofol on patients undergoing cardiac surgery. This information could provide a basis for evidence-based medicine in clinical practice.

## Methods

### Search strategy

We applied the PRISMA guidelines for the reporting of systematic reviews and meta-analyses to carry out this meta-analysis [14].

We retrieved literature on the effects of sevoflurane or propofol on myocardial protection by searching MEDLINE, the Cochrane Library, and EMBASE databases from their inception to June 2014. We supplemented this work with manual searches and reference backtracking. The keywords that were used for searching were “sevoflurane”, “propofol”, “total intravenous anesthesia”, “cardiac surgery”, “cardioprotection”, and “randomized controlled clinical trials”.

### Inclusion criteria

The following inclusion criteria were used for potentially relevant studies: (1) the participants in a study were adult patients undergoing cardiac surgery; (2) the study was a prospective, randomized, controlled clinical trial; (3) for anesthesia treatments in which the experimental group was anesthetized using sevoflurane, no propofol was used throughout the entire anesthesia process (including induction and maintenance phase), while the control group was anesthetized using propofol and no sevoflurane was used during the entire anesthesia process; (4) the study comprised detailed information, such as the number of cases, the number of controls, and the number of completed trials; and (5) the study involved measurement indices, including the postoperative cardiac index (CI), cardiac output (CO), postoperative cardiac troponin I (cTnI), postoperative mechanical ventilation time, intensive care unit (ICU) observation time, hospital stay, postoperative inotropic and vasoconstrictor drugs, postoperative atrial fibrillation, and myocardial infarction.

### Data extraction and quality assessment

Based on the predefined standard form, we abstracted the following information: the number of cases, type of anesthetic, dose of anesthetic, anesthetic method, and measurement indices.

The Jadad scale [15] evaluation system was used to assess the quality of the identified literature, based on study design, interventions, and measurement indices. Two researchers performed the evaluation independently. Disagreement was resolved through discussion with a third investigator. Any study with a Jadad score ≥ 3 was regarded as high quality.

### Statistical analysis

Review Manager (RevMan) Version 5.0 (Copenhagen: The Nordic Cochrane Centre, The Cochrane Collaboration, 2014) software was used for meta-analysis and forest plots. The weighted mean difference (WMD) with the corresponding 95 % confidence interval (CI) was calculated as the effect size for estimating numerical variables, while odds ratios (ORs) with 95 % CIs were used for dichotomous variables. The chi-square test and I^2^ statistic were performed to determine heterogeneity among studies. If significant homogeneity existed (P > 0.1 and I^2^ < 50 %), a fixed effects model was used to calculate the pooled WMD or OR. A random effects model was used if *P* < 0.1 and I^2^ > 50 %. Subgroup analysis was performed, stratified either by the type of cardiac surgery (CABG or aortic valve replacement [AVR]) or use of cardiopulmonary blood bypass during CABG (on-pump or off-pump).

### Publication bias

Publication bias detection was conducted through symmetry of funnel plots that were generated by RevMan 5.0.

### Sensitivity analysis

Sensitivity analysis was performed by calculating the pooled effect size after removing studies one at a time to evaluate whether the result would be influenced by a single study.

## Results

### Selection of studies

**Fig. 1:**
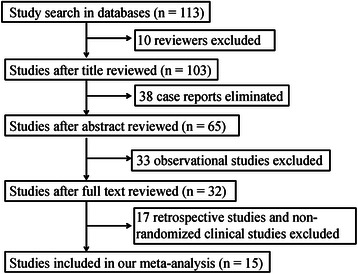
Flow chart of literature selection

Duplicated publications were removed and studies that did not involve comparison of propofol and sevoflurane were excluded. If a cohort of studies was published based on one data set, the study that had the most comprehensive data was included for the meta-analysis. As a result, a total of 113 studies were obtained through preliminary screening. Ten reviews were then excluded by browsing the title and reading the abstract. Next, 38 case reports and 33 observational studies were eliminated through further selection. From the remaining 32 studies, 17 retrospective studies and non-randomized clinical studies were removed after full text reading. Finally, 15 eligible studies [16–30] were included for the meta-analysis. A flow chart of the literature selection is shown in Fig. 1.

### Characteristics of the eligible studies and quality assessment

The characteristics of the included studies are shown in Table 1. A total of 1646 participants (1094 in the experimental group and 552 in the control group) were involved in the studies. All of the studies were published in English and they were carried out in 11 countries or regions. An assessment of quality is shown in Table 2. Because of the large proportion of high-quality research (3 studies with a score of 5, 1 study with score of 4, 3 studies with a score of 3, 5 studies with a score of 2, and 3 studies with a score of 1), the overall quality of the included studies was relatively high.

**Table 1:** Basic Information of the 15 studies included in the meta-analysis

Study	Country	Surgery	Number of cases (Sevoflurane/propofol, n)	Anesthesia			
				Sevoflurane group		Propofol group	
				Induction	Maintenance	Induction	Maintenance
Gravel et al. [10]	Canada	CABG (on-pump and off-pump)	15/15	4 % sevoflurane + 0.5 μg/kg sufentanil	0.5-2MAC sevoflurane + 0.5 μg/kg/h sufentanil	1 mg midazolam + 0.5 μg/kg sufentanil	40-150 μg/kg/min propofol + 0.5 μg/kg/h sufentanil
De Hert et al. [11]	Belgium	CABG (on-pump)	10/10	4 % sevoflurane + 0.4 μg/kg/min remifentanil	0.5–2 % sevoflurane + 0.3–0.6 μg/kg/min remifentanil	2 mg/ml propofol + 0.4 μg/kg/min remifentanil	2-4 mg/ml propofol + 0.3-0.6 μg/kg/min remifentanil
Conzen et al. [12]	Germany	CABG (off-pump)	10/10	0.3 mg/kg etomidate	2 % sevoflurane	2 μg/ml propofol	2-3 μg/ml propofol
De Hert et al. [13]	Belgium	CABG (on-pump)	15/15	2–8 % sevoflurane + 0.4 μg/kg/min remifentanil	0.5–2%sevoflurane + 0.3–0.6 μg/kg/min remifentanil	2 μg/ml propofol + 0.4 μg/kg/min remifentanil	2-4 μg/ml propofol + 0.3-0.6 μg/kg/min remifentanil
De Hert et al. [14]	Belgium	CABG (on-pump)	80/80	0.1 mg/kg midazolam + 0.4 μg/kg/min remifentanil	0.5–2 % sevoflurane + 0.2–0.4 μg/kg/min remifentanil	2 μg/mlpropofol + 0.4 μg/kg/min remifentanil	2-4 μg/ml propofol + 0.2-0.4 μg/kg/min remifentanil
Parker et al. [15]	Australia	CABG (on-pump)	118/118	10 μg/kg fentanyl + 0.1 mg/kg diazepam + 0.15 mg/kg pancuronium bromide	1–4 % sevoflurane	10 μg/kg fentanyl + 0.1 mg/kg diazepam + 0.15 mg/kg pancuronium bromide	1-8 μg/ml propofol
Kawamura et al. [16]	Japan	CABG (on-pump)	13/10	10 μg/kg fentanyl + 2–3 mg midazolam	0.5 %-1 % sevoflurane + 30 μg/kg fentanyl	10 μg/kg fentanyl + 2–3 mg midazolam	2-8 mg/kg/h propofol + 30 μg/kg fentanyl
Cromheecke et al. [17]	Belgium	AVR	15/15	0.5–1 % sevoflurane + 0.4 μg/kg/min remifentanil	0.5–1 % sevoflurane + 0.2–0.4 μg/kg/min remifentanil	2 μg/ml propofol + 0.4 μg/kg/min remifentanil	2-4 μg/mlpropofol + 0.2-0.4 μg/kg/min remifentanil
Lorsomradee et al.[18]	Belgium	CABG (on-pump)	160/160	sevoflurane + 0.2–0.4 μg/kg/min remifentanil	0.5–2 % sevoflurane + 0.2–0.4 μg/kg/min remifentanil	2 μg/ml propofol + 0.2-0.4 μg/kg/min remifentanil	2-4 μg/ml propofol + 0.2-0.4 μg/kg/min remifentanil
Law-Koune et al. [19]	France	CABG (off-pump)	9/9	8 % sevoflurane + 2 ng/mL remifentanil	sevoflurane(BIS 40–60) + remifentanil	2 μg/ml propofol + 2 ng/mL remifentanil	propofol(BIS 40–60) + remifentanil
Lucchinetti et al. [20]	Switzerland	CABG (off-pump)	10/10	fentanyl + midazolam	sevoflurane + fentanyl + midazolam	fentanyl + midazolam	propofol + fentanyl + midazolam
Yildirim et al. [21]	Turkey	CABG (on-pump)	20/20	2–8 % sevoflurane + 0.4 μg/kg/min remifentanil	0.5–2 % sevoflurane + 0.3–0.6 μg/kg/min remifentanil	0.2 μg/ml propofol + 0.4 μg/kg/min remifentanil	2-4 mg/mlpropofol + 0.3-0.6 μg/kg/min remifentanil
Ballester et al. [22]	Spain	CABG (off-pump)	18/20	0.1 mg/kg midazolam + 2–4 μg/kg fentanyl + 0.3 mg/kg etomidate	1.5–2.5 % sevoflurane	0.1 mg/kg midazolam + 2–4 μg/kg fentanyl + 0.3 mg/kg etomidate	6-8 mg/kg/h propofol
Jovic et al. [23]	Serbian	AVR	11/11	0.3 mg/kg midazolam + 0.7–1 mcg/kg sufentanil	0.1–0.2 mcg/kg/h sevoflurane	1–1.5 mg/kg propofol + 0.7–1 mcg/kg sufentanil	6-10 mg/kg/h propofol + 0.1-0.2 mcg/kg/h sufentanil
Suryaprakash et al. [24]	India	CABG (off-pump)	48/39	5–10 μg/ kg fentanyl + 0.02 mg/kg midazolam	1–2 % sevoflurane + 1 μg/kg/h fentanyl	5–10 μg/ kg fentanyl + 0.02 mg/kg midazolam	2-4 mg/kg/h propofol + 1 μg/kg/h fentanyl

*CABG* coronary artery bypass graphing, *AVR* aortic valve replacement

**Table 2:** Jadad score of the included studies

Study	Random method	Blind	Exit in follow-up	Jadad score
Gravel et al. [10]	Yes with description	Single	No	3
De Hert et al. [11]	Yes without description	Not described	No	2
Conzen et al. [12]	Yes without description	Not described	No	2
De Hert et al. [13]	Yes without description	Not described	Yes with description	2
De Hert et al. [14]	Yes with description	Double	No	5
Parker et al. [15]	Yes without description	Double	Yes with description	4
Kawamura et al. [16]	Yes without description	Single	Not described	1
Cromheecke et al. [17]	Yes with description	Not described	No	3
Lorsomradee et al. [18]	Yes with description	Double	No	5
Law-Koune et al. [19]	Yes without description	Single	Not described	1
Lucchinetti et al. [20]	Yes without description	Not described	Not described	1
Yildirim et al. [21]	Yes with description	Double	No exit	5
Ballester et al. [22]	Yes with description	Single	Yes with description	3
Jovic et al. [23]	Yes without description	Not described	No	2
Suryaprakash [24]	Yes with description	Not described	Not described	2

### Outcome of the effect of sevoflurane and propofol on cardioprotection

#### CI

**Fig. 2:**
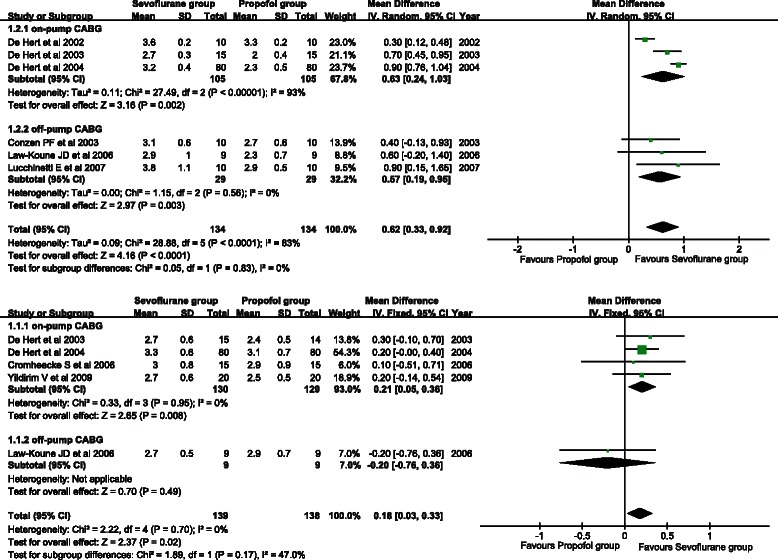
Forest plots of the postoperative cardiac index (**a**) and postoperative 12-h cardiac index (**b**). Sevoflurane and propofol groups were compared

Six studies involved the postoperative CI. A random effects model was adopted because remarkable heterogeneity existed across studies (*P* < 0.001, I^2^ = 83 %). The sevoflurane group showed a significantly higher postoperative CI than the propofol group (WMD = 0.62, 95 % CI: 0.33 to 0.92; *P* < 0.001) (Fig. 2a). Subgroup analysis showed that a higher postoperative CI was observed in the on-pump CABG subgroup (WMD = 0.63, 95 % CI: 0.24 to 1.03; *P* < 0.001) and off-pump CABG subgroup (WMD = 0.57, 95 % CI: 0.19 to 0.95; *P* = 0.003).

Five studies described the postoperative 12-h CI. A fixed effects model was used because there was no between-study heterogeneity (*P* = 0.70, I^2^ = 0 %). The postoperative 12-h CI of the sevoflurane group was significantly higher than that of the propofol group (WMD = 0.18, 95 % CI: 0.03 to 0.33; *P* = 0.02) (Fig. 2b). A similar conclusion was obtained in the on-pump CABG subgroup (WMD = 0.21, 95 % CI: 0.05 to 0.36; *P* = 0.008), but not in the off-pump CABG subgroup (WMD = −0.20, 95 % CI: −0.76 to 0.36; *P* = 0.49).

#### Cardiac output

**Fig. 3:**
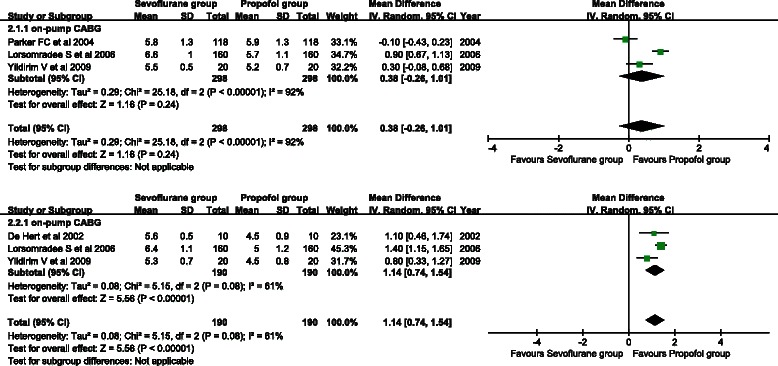
Forest plots of postoperative cardiac output (**a**) and postoperative 12-h cardiac output (**b**). Sevoflurane and propofol groups were compared

Three studies provided CO. A random effects model was used for detection of substantial heterogeneity (*P* = 0.08, I^2^ = 61 %). As shown in Fig. 3a, CO of the sevoflurane group was significantly higher than that of the propofol group (WMD = 1.14, 95 % CI: 0.74 to 1.54; *P* < 0.001).

Three studies provided postoperative 12-h CO. A random effects model was used because there was between-study heterogeneity (*P* < 0.001, I^2^ = 92 %). No significant difference in postoperative 12-h CO was found between the sevoflurane group and the propofol group (WMD = 0.38, 95 % CI: −0.26 to 1.01; *P* = 0.24) (Fig. 3b).

#### *cTnI*

**Fig. 4:**
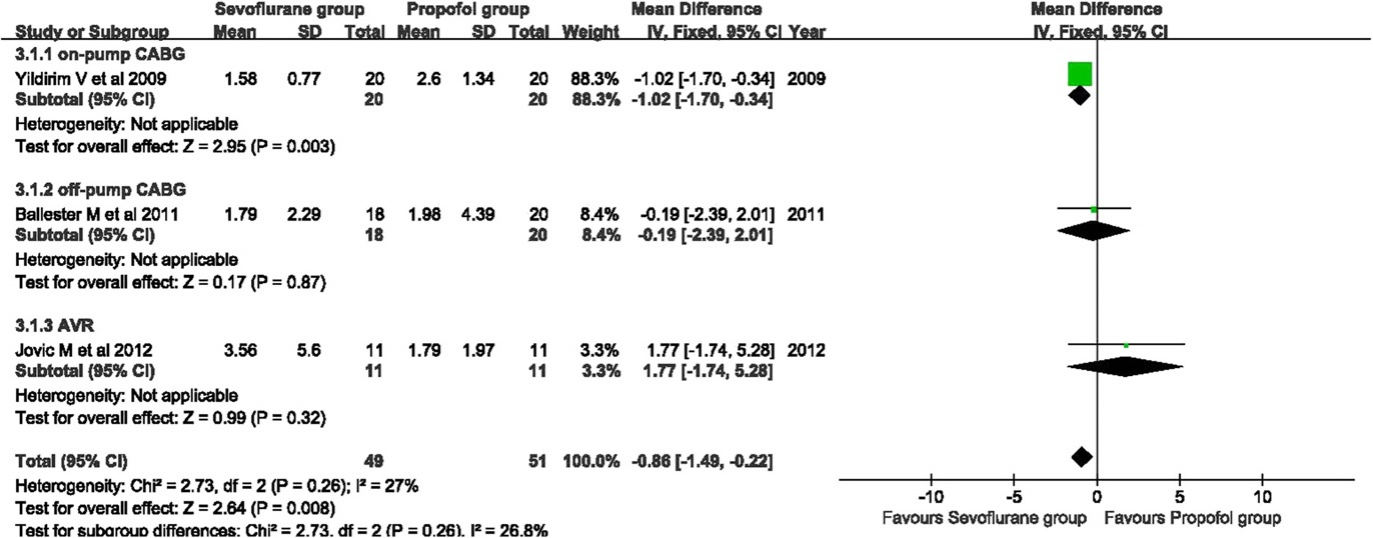
Forest plot showing comparison of postoperative 24-h cTnI between the sevoflurane and propofol groups

cTnI is an indicator of postoperative myocardial injury. Three studies provided postoperative 24-h cTnI data. Three types of surgical procedures that were used were on-pump CABG, off-pump CABG, and AVR. A fixed effects model was applied for the absence of heterogeneity across studies (*P* = 0.26, I^2^ = 27 %). As a result, sevoflurane showed significantly lower postoperative 24-h cTnI levels compared with propofol treatment (WMD = −0.86, 95 % CI: −1.49 to −0.22; *P* = 0.008) (Fig. 4).

### Mechanical ventilation time

**Fig. 5:**
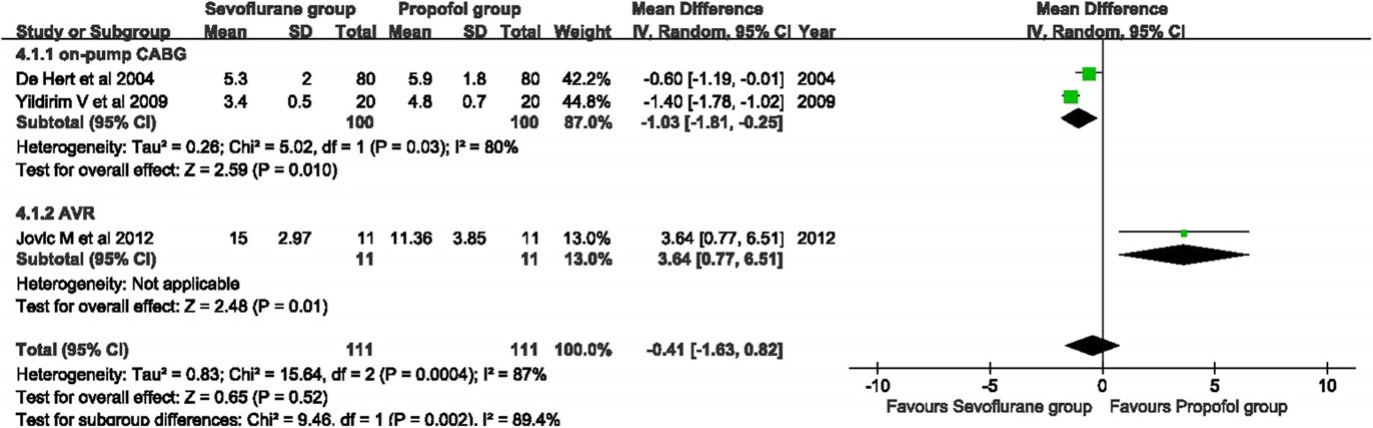
Forest plot showing comparison of mechanical ventilation time between the sevoflurane and propofol groups

Three studies reported postoperative mechanical ventilation time. A random effects model was used because of remarkable heterogeneity (*P* = 0.01, I^2^ = 77 %). There was no significant difference between the sevoflurane and propofol groups (WMD = −0.80, 95 % CI: −1.71 to 0.11; P = 0.08) (Fig. 5). Subgroup analysis showed that the mechanical ventilation time of the sevoflurane group was significantly shorter than that of the propofol group in the on-pump CABG subgroup (WMD = −1.03, 95 % CI: −1.81 to −0.25; *P* = 0.010). However, no significant difference was observed in the AVR subgroup (WMD = 1.64, 95 % CI: −1.23 to 4.51; *P* = 0.26).

### Drug use

**Fig. 6:**
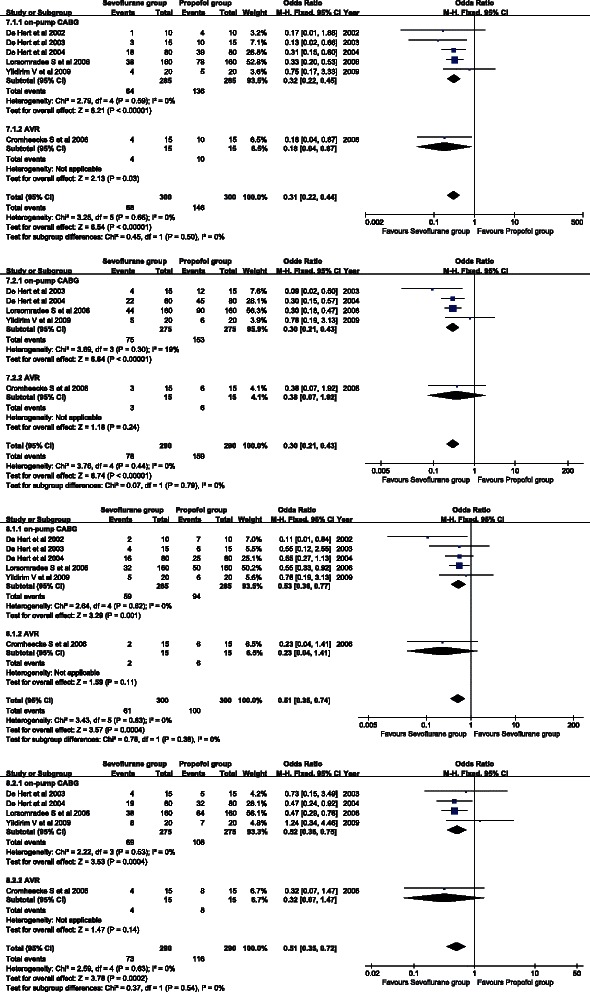
Forest plot of postoperative inotropic drug use (**a**). Forest plot of inotropic drug use during the ICU stay (**b**). Forest plot of postoperative vasoconstrictor drug use (**c**). Forest plot of vasoconstrictor drug use during ICU stay (**d**). Sevoflurane and propofol groups were compared

Six studies reported postoperative inotropic drug use data. A fixed effects model was adopted there was no heterogeneity between studies (*P* = 0.66, I^2^ = 0 %). Inotropic drug use of the sevoflurane group was significantly less than that of the propofol group (OR = 0.31, 95 % CI: 0.22 to 0.44; *P* <0.001) (Fig. 6a). A similar conclusion was obtained in the on-pump CABG subgroup (OR = 0.32, 95 % CI: 0.22 to 0.45; *P* < 0.001) and the AVR subgroup (OR = 0.18, 95 % CI: 0.04 to 0.36; P = 0.03).

Five studies provided inotropic drug use data during the ICU stay. A fixed effects model was used for the homogeneity (*P* = 0.44, I^2^ = 0 %). Inotropic drug use during the ICU stay of the sevoflurane group was significantly less than that of the propofol group (OR = 0.30, 95 % CI: 0.21 to 0.43; *P* < 0.001) (Fig. 6b). A similar conclusion was obtained in the on-pump CABG subgroup (OR = 0.30, 95 % CI: 0.21 to 0.43; *P* < 0.001), but not in the AVR subgroup (OR = 0.38, 95 % CI: 0.07 to 1.92; *P* = 0.24).

Six studies contained information about postoperative vasoconstrictor drug use. A fixed effects model was applied for the absence of heterogeneity between studies (*P* = 0.63, I^2^ = 0 %). Postoperative vasoconstrictor drug use of the sevoflurane group was significantly lower than that of the propofol group (OR = 0.51, 95 % CI: 0.35 to 0.74; *P* = 0.0004) (Fig. 6c). A similar conclusion was obtained in the on-pump CABG subgroup (OR = 0.53, 95 % CI: 0.36 to 0.77; *P* = 0.001), but not in the AVR subgroup (OR = 0.23, 95 % CI: 0.04 to 1.41; *P* = 0.11).

Five studies reported information about vasoconstrictor drug use during the ICU stay. There was no heterogeneity between studies (*P* = 0.63, I^2^ = 0 %); therefore, a fixed effects model was applied. Vasoconstrictor drug use during the ICU stay of the sevoflurane group was significantly less than that of the propofol group (OR = 0.51, 95 % CI: 0.35 to 0.72; *P* = 0.0002) (Fig. 6d). A similar conclusion was obtained in the on-pump CABG subgroup (OR = 0.52, 95 % CI: 0.36 to 0.75; *P* = 0.0004), but not in the AVR subgroup (OR = 0.32, 95 % CI: 0.07 to 1.47; *P* = 0.14).

### Postoperative ICU length of stay

**Fig. 7:**
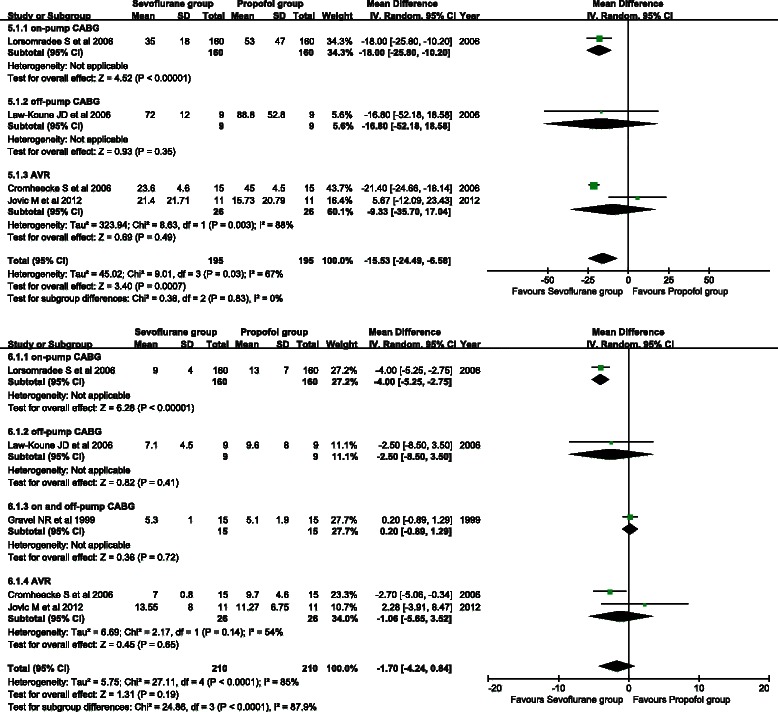
Forest plots of postoperative ICU length of stay (**a**) and hospital length of stay (**b**). Sevoflurane and propofol groups were compared

Four studies involved postoperative length of stay in the ICU. A random effects model was applied because of between-study heterogeneity (*P* = 0.03, I^2^ = 67 %). The postoperative ICU length of stay was considerably lower in the sevoflurane group than in the propofol group (WMD = −15.53, 95 % CI: −24.29 to −6.58; *P* = 0.0007) (Fig. 7a). A similar conclusion was obtained in the on-pump CABG subgroup (WMD = −18.00, 95 % CI: −25.80 to −10.20; P < 0.001), but not in the other subgroups.

Five studies reported postoperative hospital stay data. A random effects model was adopted because of between-study heterogeneity (*P* < 0.001, I^2^ = 85 %). There was no significant difference in postoperative length of hospital stay between the two groups (WMD = −1.70, 95 % CI: −4.24 to 0.84; *P* = 0.19) (Fig. 7b). Subgroup analysis of the on-pump CABG subgroup showed that the length of hospital stay of the sevoflurane group was significantly shorter than that of the propofol group (WMD = −4.00, 95 % CI: −5.25 to −2.75; *P* < 0.001). No significant difference was found in hospital length of stay in the other subgroups.

### Incidence of postoperative complications and mortality

**Fig. 8:**
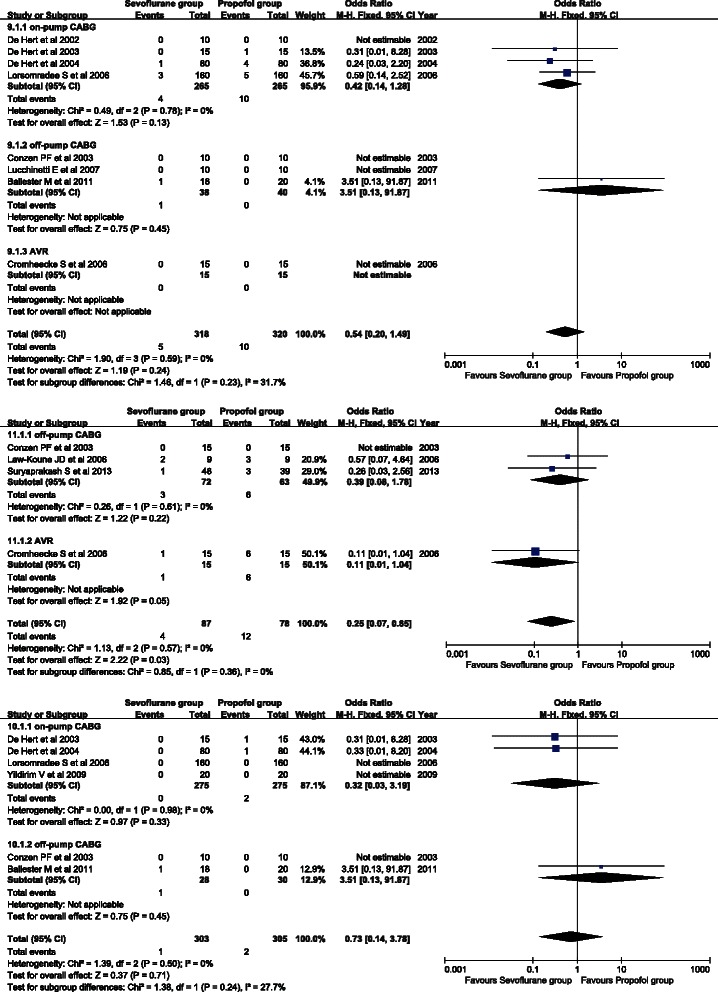
Forest plots of the incidence of postoperative myocardial infarction (**a**). Forest plot of atrial fibrillation (**b**). Forest plot of mortality (**c**). Sevoflurane and propofol groups were compared

Eight studies provided information on the incidence of postoperative myocardial infarction. A fixed effects model was used for observation of homogeneity (*P* = 0.59, I^2^ = 0 %). There was no significant difference in the incidence of postoperative myocardial infarction between the sevoflurane and propofol groups (OR = 0.54, 95 % CI: 0.20 to 1.49; *P* = 0.24) (Fig. 8a). There was also no significant difference in any of the subgroups.

Five studies provided the incidence of postoperative atrial fibrillation. No between-study heterogeneity was detected (*P* = 0.57, I^2^ = 0 %) and a fixed effects model was used. The incidence of postoperative atrial fibrillation of the sevoflurane group was significantly lower than that of the propofol group (OR = 0.25, 95 % CI: 0.07 to 0.85; *P* = 0.03) (Fig. 8b). No significant difference was found in any of the subgroups.

Six studies provided postoperative mortality data. A fixed effects model was used in analysis because there was no heterogeneity between studies (*P* = 0.50, I^2^ = 0 %). There was no significant difference in mortality between the sevoflurane and propofol groups (OR = 0.73, 95 % CI: 0.14 to 3.78; *P* = 0.71) (Fig. 8c). Subgroup analysis showed no significant difference in mortality in any of the subgroups.

### Publication bias and sensitivity analysis

**Fig. 9:**
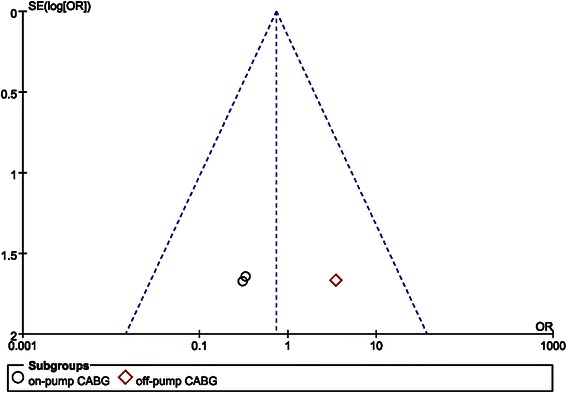
Publication bias analysis according to a funnel plot for postoperative mortality

Sensitivity analysis showed that the conclusion was not affected by exclusion of any single included study. No obvious publication bias was found according to the funnel plots (Fig. 9).

## Discussion

In this study, we retrieved 15 randomized, controlled trials to compare the cardioprotective effect of sevoflurane with propofol anesthesia in cardiac surgery. We found that sevoflurane was superior to propofol in the postoperative CI, postoperative 24-h CI, postoperative CO, postoperative 24-h cTnI concentrations, inotropic drug use, vasoconstrictor drug use, ICU length of stay, and incidence of atrial fibrillation. However, no significant difference was found in postoperative 12-h CO, mechanical ventilation time, hospital length of stay, incidence of myocardial infarction, and mortality between sevoflurane and propofol. All of these findings were consistent with a previous meta-analysis, except for inotropic drug use and the incidence of atrial fibrillation, which showed a comparable effect [13]. However, subgroup analysis was not involved in this previous study and the samples were relatively small. By contrast, our study applied a more strict inclusion criterion in case and control experimental design. We focused only on studies in which the experimental group was anesthetized using sevoflurane but not propofol throughout the entire anesthesia process, while the control group was anesthetized by propofol but not sevoflurane. In Yao and Li’s meta-analysis [13], the experimental or control group was anesthetized using both sevoflurane and propofol in some of the included studies. Additionally, we carried out subgroup analysis in our study, which made the results more precise than previous analyses.

The level of cTnI is a sensitive and specific marker of myocardial injury. A sevoflurane-induced reduction in cTnI levels is associated with a lower incidence of late adverse cardiac events [31]. Accordingly, we observed a lower incidence of atrial fibrillation in the sevoflurane group than in the propofol group. ICU and the hospital stay length are two comprehensive indicators that are associated with postoperative complications and medical fees. The current study showed that use of sevoflurane led to a shorter ICU stay length, but not hospital stay length compared with the propofol group. This finding might be explained by different study populations and varying hospital operating standards. Moreover, reduced inotropic drug use in the sevoflurane group is consistent with the study by Yu et al. [8], which showed that volatile anesthetics benefit myocardial energy stores during ischemia and subsequent recovery after reperfusion [8].

Volatile anesthetics have a long-lasting cardioprotective effect that enhances their administration during cardiac surgery [32]. Extensive studies have confirmed that sevoflurane protests against ischemic myocardial damage [32, 33]. However, in our study, no significant difference was detected in the incidence of postoperative myocardial infarction between sevoflurane and propofol, suggesting that propofol might have comparable myocardial protection with sevoflurane.

In our meta-analysis, all of the included 15 studies were prospective, randomized, clinical trials, and their quality was high. Additionally, we conducted subgroup analysis to obtain accurate results. However, several limitations should be mentioned. First, because of language restrictions and database updates, we could not include literature in other languages nor those yet to be published. Including those studies would have affected our results. Second, the sample size was small and no blinding was mentioned in some studies. Third, differences in surgical and medical technology among the studies included also affected the results. Finally, significant heterogeneity was observed in some assessment indices, which might have caused bias in our results. We assert that more high-quality, randomized, controlled trials are warranted.

## Conclusions

In conclusion, sevoflurane anesthesia has a better cardioprotective effect on patients undergoing cardiac surgery according to several indicators than propofol anesthesia. However, multicenter, prospective, randomized, controlled trails with large samples, uniform criteria, and surgical procedures are necessary to further confirm the advantage of sevoflurane over propofol.
